# Preventive Behaviors Conveyed on YouTube to Mitigate Transmission of COVID-19: Cross-Sectional Study

**DOI:** 10.2196/18807

**Published:** 2020-04-02

**Authors:** Corey H Basch, Grace C Hillyer, Zoe C Meleo-Erwin, Christie Jaime, Jan Mohlman, Charles E Basch

**Affiliations:** 1 William Paterson University Wayne, NJ United States; 2 Mailman School of Public Health Columbia University New York, NY United States; 3 Teachers College Columbia University New York, NY United States

**Keywords:** YouTube, COVID-19, social media, pandemic, outbreak, infectious disease, public health, prevention

## Abstract

**Background:**

Accurate information and guidance about personal behaviors that can reduce exposure to severe acute respiratory syndrome coronavirus 2 are among the most important elements in mitigating the spread of coronavirus disease 2019 (COVID-19). With over 2 billion users, YouTube is a media channel that millions turn to when seeking information.

**Objective:**

At the time of this study, there were no published studies investigating the content of YouTube videos related to COVID-19. This study aims to address this gap in the current knowledge.

**Methods:**

The 100 most widely viewed YouTube videos uploaded throughout the month of January 2020 were reviewed and the content covered was described. Collectively, these videos were viewed over 125 million times.

**Results:**

Fewer than one-third of the videos covered any of the seven key prevention behaviors listed on the US Centers for Disease Control and Prevention website.

**Conclusions:**

These results represent an important missed opportunity for disease prevention.

## Introduction

In December 2019, several cases of pneumonia of an unknown etiology were reported in Wuhan, China [1]. On January 20, 2020, the Centers for Disease Control and Prevention (CDC) as well as state and local health departments began monitoring the burgeoning situation [2]. By the end of January, the World Health Organization (WHO) had declared the outbreak to be of serious concern [3]. The disease, now known as coronavirus disease 2019 (COVID-19) and caused by severe acute respiratory syndrome coronavirus 2 (SARS-CoV-2), was officially named by WHO on February 11, 2020 [4]. By March 7, 2020, the global number of reported cases had surpassed 100,000 [5].

As a novel global health threat, the scientific community has only begun to investigate the distinguishing features of COVID-19. Epidemiological and biomedical research thus far suggests the following. The incubation period ranges from 1-14 days [6]. COVID-19 is largely spread by contact with respiratory droplets from an infected individual. Symptoms most commonly include fever, fatigue, and a dry cough [6]. The Chinese Center for Disease Control and Prevention reports from mid-February indicated a case fatality rate of 2.3% and that 81% of cases were mild in nature [7]. It is understood that the case fatality rate will shift as more cases are identified. However, it also became clear that COVID-19 is substantially deadlier than seasonal influenza [8]. The risk of severe illness and death appears to be concentrated among older populations and those with underlying medical conditions [9].

It is unclear how long SARS-CoV-2 survives on surfaces, but estimates suggest anywhere from a few hours to several days [10]. Community spread has been reported worldwide [3], and on March 11, 2020, the WHO declared COVID-19 a pandemic [11]. Testing in the United States was delayed in late February and March, hampering case identification efforts [12]. Testing may have been hindered initially by not only a lack of tests but also out-of-pocket costs for those uninsured or underinsured [13]; on March 5, 2020, America’s Health Plans indicated they would waive copays for COVID-19 testing. Containment is complicated by the fact that substantial numbers of Americans lack paid sick leave [14], and, as of March 13, 2020, public schools in densely populated urban areas such as New York continued to remain open. Projections of widespread transmission indicate that health care systems may rapidly become overwhelmed [3]. There are no specific treatments for the virus at present [9]. Concerns over COVID-19 have had a swift impact on global financial markets with potential long-term repercussions on a number of sectors [15]. As with SARS-CoV, a number of news outlets have reported a swift uptick in anti-Asian racism [16-18]. In some areas, public fears about COVID-19 have led to panic buying of supplies including personal protective equipment, reducing the availability of necessary supplies for health care workers [10].

One of the most important aspects of an effective campaign to minimize COVID-19 transmission is accurate information that is conveyed in a way that is understood by the public. Google Trends demonstrates a substantial spike in interest in COVID-19 since early February 2020 [19], and the WHO has characterized the exponential increase in information (and misinformation) about COVID-19 as an “infodemic” [20]. The CDC and WHO have steadily been posting content discoverable on the internet. Moreover, the WHO has been working with social media outlets to ensure users searching for information on COVID-19 are guided to reliable sources [21]. Despite these efforts, in novel and rapidly evolving situations, there is a high potential for misinformation and disinformation to spread through online sources [22,23]. As the second most popular social media platform after Google [24] and with over 2 billion users [25], YouTube is a media channel that millions turn to when seeking information on COVID-19. Even videos that cover COVID-19 in a particular national context likely have a global reach. Although previous investigations have examined the content of YouTube videos on infectious diseases such as Ebola [26,27], H1N1 influenza [28,29], West Nile virus [30], and Zika virus [31], we did not identify any published studies that have investigated the content of YouTube videos related to COVID-19. This study, therefore, aimed to address this gap in current knowledge.

## Methods

The sample of videos was delimited to the 100 most widely viewed YouTube videos uploaded throughout the month of January 2020. The keyword “Coronavirus” was used as the search term, which was the most widely used terminology to describe COVID-19 at that time. The videos were sorted by view count to identify the 100 most widely viewed videos (in both English, including subtitles, and Spanish). There were 7 videos excluded and replaced: 5 for irrelevance (not about the virus) and 2 because they were in a language other than English or Spanish.

Coding categories (Table 1) were created using a CDC fact sheet on COVID-19 [3,9] and prior YouTube studies on Ebola and Zika [26,31]. Content was classified into 5 categories: prevention behaviors, mortality and fear, symptoms, transmission and natural history, or other precautions. The characteristics of videos that were coded included number of views, length in minutes, language, presentation style, and source and date of upload. Three sources of upload comprised mutually exclusive and exhaustive categories: consumer, professional (MD or RN), and television- or internet-based news. Author CJ coded content across all videos, and a second author (CHB) coded a randomly selected subset of 10 videos to ascertain inter-rater reliability, which was assessed using Cohen kappa and found to be excellent (k=0.971).

Descriptive statistics were calculated, including frequencies and percentages and, where appropriate, means and standard deviations. Differences between content covered in videos uploaded from different sources was assessed by chi-square tests using a two-sided *P* value <.05. All analyses were performed using SPSS version 26 (IBM Corp).

**Table 1:** Description of content covered in 100 widely viewed YouTube videos about coronavirus disease 2019, January 2020.

Categories		Total (N=100), n (%)	Number of views (n=125,286,561), n (%)		Consumer (n=11), n (%)	Professional (n=4), n (%)	News (n=85), n (%)	*P* value
**Prevention behaviors**								
	Hand hygiene	26 (26)	33,268,243 (26.55)		4 (36)	2 (50)	20 (24)	.39
	Avoid close contact with those who are sick	31 (31)	41,269,546 (32.94)		5 (45)	3 (75)	23 (27)	.09
	Stay home when ill	29 (29)	42,647,990 (34.04)		5 (45)	2 (50)	22 (26)	.28
	Cover cough/sneeze with tissue; throw tissue away	14 (14)	19,625,830 (15.66)		3 (27)	1 (25)	10 (12)	.36
	Use facemask for protection if you are caring for the ill	0 (0)	0 (0.00)		0 (0)	0 (0)	0 (0)	N/A^a^
	Use facemask for protecting others if you are ill	2 (2)	1,152,765 (0.92)	1 (9)		0 (0)	1 (1)	.36
	Clean and disinfect highly touched objects and surfaces	16 (16)	17,545,061 (14.00)		4 (36)	2 (50)	10 (12)	.04
**Mortality or fear**								
	Mentions death	84 (84)	101,216,230 (80.79)		9 (82)	4 (100)	71 (84)	.49
	Suggests anxiety or fear	79 (79)	101,017,274 (80.63)		10 (91)	3 (75)	66 (78)	.53
**Symptoms**								
	Coughing	37 (37)	48,785,552 (38.94)		5 (45)	1 (25)	31 (36)	.74
	Shortness of breath	26 (26)	36,446,095 (29.09)		4 (36)	1 (25)	21 (25)	.72
	Fever	43 (43)	59,530,161 (47.52)		7 (64)	3 (75)	33 (39)	.12
**Transmission and natural history**								
	Modes of transmission	42 (42)	63,474,010 (50.66)		5 (45)	4 (100)	33 (39)	.025
	Incubation period	47 (47)	55,706,189 (44.46)		7 (64)	3 (75)	37 (44)	.23
	Treatment	21 (21)	33,676,717 (26.88)		2 (18)	1 (25)	18 (21)	.96
**Other precautions**								
	Quarantine	89 (89)	109,741,111 (87.59)		11 (100)	4 (100)	74 (87)	.15
	Remain indoors	39 (39)	59,527,347 (47.51)		6 (55)	2 (50)	31 (36)	.47
	Restrict travel	84 (84)	96,914,919 (77.35)		10 (91)	3 (75)	71 (84)	.71

^a^Not applicable.

## Results

At the time of data collection (January 31, 2020), the videos in the sample were viewed more than 125 million times (by March 5, 2020, these videos garnered an additional 41 million views). The mean number of views per video was 1,252,865.6 (SD 954,752.0), and the mean length was 6.4 minutes (SD 6.4 minutes; range 15 seconds to 45 minutes). Of the 100 videos, the majority (n=85, 85.0%) were uploaded by news agencies (aired on television or the internet). Most were created in English (n=72, 72.0%) or with English subtitles (n=14, 14.0%), but 14.0% (n=14) were in Spanish. The large majority (n=87, 87.0%) featured a live presenter and 13.0% (n=13) featured animation.

Fewer than one-third of the videos covered any of the seven key prevention behaviors listed on the CDC website (Table 1). Just over a quarter of the videos covered hand hygiene and less than one-fifth mentioned covering a cough or sneeze with a tissue and then discarded. Although 45.0% (n=45) mentioned using a face mask, none recommended using a face mask when caring for someone who is sick, and only 2 mentioned using a face mask if you are sick to protect others. Cleaning and disinfecting frequently touched surfaces was mentioned in less than one-fifth of the videos.

The majority of videos mentioned death, or suggested anxiety or fear. Symptoms, transmission, and natural history were covered in fewer than half of the videos. Quarantine and travel restrictions were mentioned in the majority of videos. The content covered generally did not vary by source of upload.

## Discussion

The videos in the study sample were viewed over 125 million times as of January 31, 2020 (and increased by over 30% to over 165 million views by March 5, 2020), indicating the considerable reach of YouTube as a way to communicate with the public. Knowledge about the biology, pathophysiology, and epidemiology of COVID-19 is evolving rapidly. What we do know at this time is that personal behaviors are the best way to prevent disease transmission and COVID-19.

Accurate information and guidance about personal behavior is, therefore, one of the most important elements in mitigating the spread of COVID-19. Primary prevention of any disease relies on two components: reducing exposure and reducing susceptibility. Given that no vaccine is currently available to reduce susceptibility, the most effective way to prevent disease transmission and prevent illness is by preventing exposure. COVID-19 is a propagated epidemic (spreads from person-to-person) and is thought to be transmitted through both direct and indirect contact [32]. The CDC recommends behaviors to protect individuals by reducing exposure: proper hand hygiene (including avoiding touching one’s nose, mouth, and eyes with unwashed hands) and avoiding close contact, not only with people who are sick, but also through social distancing (especially for those at higher risk, namely, older adults and those with chronic illnesses). The CDC further recommends behaviors to protect others: staying home when sick (except to receive medical care); covering one’s sneeze or cough with a tissue (or inside of elbow), then discarding the tissue in trash, immediately followed by proper hand hygiene; wearing a mask if ill when around others or caring for someone who is sick; and cleaning and disenfecting frequently touched surfaces [33]. These recommendations may be difficult to understand, especially for the considerable proportion of the public with low levels of reading literacy. Video presentation is a potentially useful alternative for communicating key information to the public. We found that fewer than one-third of the most widely viewed YouTube videos covered any of these behavioral recommendations, which we believe represents an important missed opportunity for disease prevention.

In contrast, the majority of the 100 videos mentioned number of deaths or estimated mortality rates, or suggested fear and anxiety, and these videos were collectively viewed over 100 million times. Communications that increase fear and anxiety may prompt preventive actions, but may also lead to maladaptive, socially irresponsible behaviors such as hoarding medical supplies, hygienic supplies, and food items and making unnecessary visits to physicians and emergency rooms [34]. Accurate information must be conveyed by designated spokespersons who can promote primary and secondary prevention behaviors, model rational thinking, and allay unrealistic or excessive fears about the future [35]. The most widely viewed YouTube videos on COVID-19 do not achieve these aims. Thus, we conclude that in addition to reducing risk of exposure through recommended behaviors, it is clear that consumers must also become critical evaluators of disseminated information about COVID-19 found on YouTube.

This study extends awareness about the content of widely viewed videos on YouTube during the early days of the COVID-19 outbreak, but there are limitations that must be mentioned. Recommendations are being updated frequently and more recent YouTube videos may cover preventive behaviors to a greater extent. Nevertheless, the number of views garnered by videos in our sample continued to grow. As with all cross-sectional studies, the use of one data collection point is limiting. As the state of YouTube is in a state of fluctuation, thus, the videos with the most views may change over time. It is also possible that, as more information is learned about the disease, common search terms and content will evolve, despite older videos remaining online.

It is often not possible to determine the geographic location of YouTube posters. With over 2 billion users worldwide, many users likely watch videos regardless of the national origin of posters. Given this, we cannot make claims about the regionality or lack thereof of YouTube videos on COVID-19. Nevertheless, it is fair to assert that the Spanish and English language videos on COVID-19 in this sample could be reaching viewers around the world. Additionally, the prevention behaviors noted in this study could be applicable worldwide. In addition, our sample of videos was small, and 100 was an arbitrary cut point for inclusion. Further, there are issues with the impermanence of video content in that highly viewed videos may contain outdated information regardless of the accuracy of the information when the videos were produced. In addition to efforts to promote authoritative information, YouTube could benefit from clearly demarcating the most current, valid information. This would be especially useful in instances such as the COVID-19 pandemic, where information is changing rapidly. Although this study represents this sample of videos at a point in time, it offers insight about the nature of content that is and is not covered, and suggests opportunities to convey information to mitigate the spread of COVID-19 and help people make informed decisions about caring for and protecting themselves and their families.

## Abbreviations

CDC: Centers for Disease Control and Prevention
COVID-19: coronavirus disease 2019
SARS-CoV-2: severe acute respiratory syndrome coronavirus 2
WHO: World Health Organization
