# Correction: Preventive Behaviors Conveyed on YouTube to Mitigate Transmission of COVID-19: Cross-Sectional Study

**DOI:** 10.2196/19601

**Published:** 2020-05-06

**Authors:** Corey H Basch, Grace C Hillyer, Zoe C Meleo-Erwin, Christie Jaime, Jan Mohlman, Charles E Basch

**Affiliations:** 1 William Paterson University Wayne, NJ United States; 2 Mailman School of Public Health Columbia University New York, NY United States; 3 Teachers College Columbia University New York, NY United States

The authors of “Preventive Behaviors Conveyed on YouTube to Mitigate Transmission of COVID-19: Cross-Sectional Study” (JMIR Public Health Surveill 2020;6(2):e18807), noticed the following errors in their published article.

In Table 1, in the row “Stay home when ill”, under the column “News (n=85), n (%)”, the values have been revised from “12 (14)” to “22 (26)”. In the same row, the value under the column “*P* value” has been revised from “.03” to “.28”.

This error did not have any substantive effects on the results or conclusions of the study.

Additionally, the URL in Reference 9 was inadvertently listed as a proxy. Reference 9 has been revised from:

Centers for Disease Control and Prevention. Coronavirus disease 2019 (COVID-19): prevention & treatment 2020 URL: https://www-cdc-gov.ezproxy.cul.columbia.edu/coronavirus/2019-ncov/about/prevention-treatment.html [accessed 2020-03-08]

to:

Centers for Disease Control and Prevention. Coronavirus disease 2019 (COVID-19): prevention & treatment 2020 URL: https://www.cdc.gov/coronavirus/2019-ncov/prevent-getting-sick/prevention.html [accessed 2020-03-08]

These corrections will appear in the online version of the paper on the JMIR website on May 6, 2020, together with the publication of this correction notice. Because this was made after submission to PubMed, PubMed Central, and other full-text repositories, the corrected article has also been resubmitted to those repositories.

